# Altered Cerebrospinal Fluid Clearance and Increased Intracranial Pressure in Rats 18 h After Experimental Cortical Ischaemia

**DOI:** 10.3389/fnmol.2021.712779

**Published:** 2021-08-09

**Authors:** Steven W. Bothwell, Daniel Omileke, Rebecca J. Hood, Debbie-Gai Pepperall, Sara Azarpeykan, Adjanie Patabendige, Neil J. Spratt

**Affiliations:** ^1^The School of Biomedical Sciences and Pharmacy, The University of Newcastle, Newcastle, NSW, Australia; ^2^Hunter Medical Research Institute, Newcastle, NSW, Australia; ^3^Institute of Infection, Veterinary & Ecological Sciences, University of Liverpool, Liverpool, United Kingdom; ^4^Hunter New England Local Health District, Newcastle, NSW, Australia

**Keywords:** cerebrospinal fluid, infarct expansion, neurological deterioration, ischaemia, intracranial pressure, stroke, lymphatics, spinal clearance

## Abstract

Oedema-independent intracranial pressure (ICP) rise peaks 20–22-h post-stroke in rats and may explain early neurological deterioration. Cerebrospinal fluid (CSF) volume changes may be involved. Cranial CSF clearance primarily occurs *via* the cervical lymphatics and movement into the spinal portion of the cranio-spinal compartment. We explored whether impaired CSF clearance at these sites could explain ICP rise after stroke. We recorded ICP at baseline and 18-h post-stroke, when we expect changes contributing to peak ICP to be present. CSF clearance was assessed in rats receiving photothrombotic stroke or sham surgery by intraventricular tracer infusion. Tracer concentration was quantified in the deep cervical lymph nodes *ex vivo* and tracer transit to the spinal subarachnoid space was imaged *in vivo*. ICP rose significantly from baseline to 18-h post-stroke in stroke vs. sham rats [median = 5 mmHg, interquartile range (IQR) = 0.1–9.43, *n* = 12, vs. −0.3 mmHg, IQR = −1.9–1.7, *n* = 10], *p* = 0.03. There was a bimodal distribution of rats with and without ICP rise. Tracer in the deep cervical lymph nodes was significantly lower in stroke with ICP rise (0 μg/mL, IQR = 0–0.11) and without ICP rise (0 μg/mL, IQR = 0–4.47) compared with sham rats (4.17 μg/mL, IQR = 0.74–8.51), *p* = 0.02. ICP rise was inversely correlated with faster CSF transit to the spinal subarachnoid space (*R* = −0.59, *p* = 0.006, Spearman’s correlation). These data suggest that reduced cranial clearance of CSF via cervical lymphatics may contribute to post-stroke ICP rise, partially compensated *via* increased spinal CSF outflow.

## Introduction

Ischaemic stroke is a leading cause of death and disability worldwide (Mathers and Loncar, 2006). Stroke severity is variable, and some patients present with relatively minor stroke. While occlusion of small branch vessels accounts for many of those with minor symptoms, it is now recognised that some patients with minor symptoms may have large vessel occlusion (Smith et al., 2005). Around 10–20% of patients with minor stroke or transient ischaemic attack will experience early recurrent stroke and infarct expansion, and the vast majority of these occur in the initially ischaemic vascular territory (Asdaghi et al., 2011; Coutts et al., 2012).

The most likely cause of infarct expansion is failure of leptomeningeal collateral vessels. Failure of initially good collateral blood flow is associated with infarct growth following ischaemic stroke (Campbell et al., 2013). The cause of collateral vessel failure has not been definitively established. However, after stroke, blood flow in these vessels is largely driven by cerebral perfusion pressure, which is sensitive to changes in intracranial pressure (ICP) (Czosnyka and Pickard, 2004). In support of this, our previous work showed that elevation of ICP during middle cerebral artery occlusion (MCAo) in rats caused a linear reduction of collateral blood flow (Beard et al., 2015). We previously identified a dramatic rise in ICP present at 24 h after minor ischaemic stroke in rats, which peaked at 20–22 h (Murtha et al., 2014a; Beard et al., 2016). Other labs have shown that ICP is elevated around 24 h post-stroke in both small and large experimental ischaemia (Kotwica et al., 1991; Silasi et al., 2009). Clinical observations of stroke patients also found elevated ICP at 24 h (Kovacs et al., 2017). The time point of this ICP rise, taken with our understanding of how ICP influences collateral blood flow, suggests a possible mechanism of collateral failure and infarct expansion.

Our understanding of the underlying mechanisms of this ICP rise is limited. Tissue, blood, and cerebrospinal fluid (CSF) volume all determine ICP. We know that oedema (tissue volume) is not the primary cause (Murtha et al., 2015) and pilot data from our lab suggest that there is no contribution of cerebral blood volume (McLeod et al., 2014). Emerging evidence suggests that CSF volume is increased post-stroke. Resistance to CSF outflow is increased 18 h post-stroke in a rat model of cortical ischaemia and 24 h post-stroke in a model of striatal stroke (Patabendige et al., 2019; Alshuhri et al., 2020). These studies indicate that increased CSF volume may contribute to ICP rise after stroke.

We are only now beginning to understand the importance and complexities of CSF dynamics (Bothwell et al., 2019). Traditional textbook explanations of CSF clearance describe the movement of CSF into the superior sagittal sinus *via* arachnoid projections (Weed, 1914; Davson and Segal, 1996). However, our understanding has now shifted in line with a large body of work that describes CSF drainage from the subarachnoid space to the deep cervical lymphatics in multiple species (Boulton et al., 1999; Mollanji et al., 2001a, b; Mathieu et al., 2013; Ma et al., 2017; Maloveska et al., 2018). There are two primary mechanisms hotly debated in contemporary discussions of CSF clearance to the lymphatics system. The first describes the clearance of CSF from the subarachnoid space into the nasal mucosa along the perineural sheathes of olfactory nerves (Faber, 1937; Jackson et al., 1979; Walter et al., 2006; Ma et al., 2017; Norwood et al., 2019). These nerves cross the cribriform plate and once in the nasal mucosa, CSF moves to lymphatic vessels of the submucosa and onto the deep cervical lymphatics (Bradbury and Westrop, 1983; Zakharov et al., 2004; Proulx, 2021). The second mechanism describes the uptake of CSF into lymphatic vessels located in the dura mater, described as the “meningeal lymphatics,” with some evidence that the basal cisterns are hotspots of CSF uptake (Aspelund et al., 2015). In this model, the meningeal lymphatics support direct clearance of CSF into the deep cervical lymphatics that is distinct from lymphatic uptake at the nasal mucosa (Louveau et al., 2015). Either way, clearance is directed to the cervical lymphatics.

Our lab previously implicated CSF clearance into the nasal mucosa as a major outflow pathway in rats, along with drainage into the spinal canal (Murtha et al., 2014b). In rodents, CSF has been shown to travel along the spinal subarachnoid space and canal before moving into lymphatic vessels of the sacral spine (Ma et al., 2019). In this study, we aimed to determine whether cranial CSF clearance is altered in an animal model of ischaemic stroke, and whether alterations are likely to contribute to ICP rise after stroke.

## Materials and Methods

### Animals

Procedures were carried out on male outbred Wistar rats aged between 8 and 12 weeks (*n* = 31) weighing between 280 and 320 g. All experimental animal procedures used in this project were in accordance with the Australian Code of Practice for the Care and Use of Animals for Scientific Purposes and were approved by the Animal Care and Ethics Committee of the University of Newcastle (A-2013-343).

Animals were excluded from experiments if they presented with congenital deformities that obstructed surgical laminectomy to expose the imaging window, or if technical errors preventing accurate penetration of the ventricles and infusion of Evans blue dye occurred. Animals were only included in the final analysis after confirmation of stroke, and ventricle penetration of infusion catheter by histology.

Animals were assigned to stroke or sham groups prior to intervention. Blinding was not possible during experimental procedures; however, the investigator was blinded at the time of data extraction and analysis: each animal was assigned an experiment number and analysis was carried out on all data in one sitting with no indication of experimental stroke status.

### Anaesthesia and Monitoring

Rats were anaesthetised with isoflurane (5% induction, 2–2.5% maintenance) in 50:50% N_2_:O_2_. Incision sites were injected subcutaneously with 2 mg/kg 0.05% Bupivacaine (Pfizer, Sydney, Australia). Core body temperature was regulated and maintained at 37°C *via* a thermocouple rectal probe (RET-2, Physitemp Instruments Inc., Clifton, NJ, United States) and heat mat. Blood gases and pH were measured in a fast blood analyser (i-STAT 1; Abbott, Australia) at baseline prior to stroke and prior to Evans blue dye infusion from 0.1 mL blood samples taken from a femoral arterial line. This line was also used for arterial blood pressure monitoring. Prior to recovery, an additional Bupivacaine injection (0.3 mL, 0.05%, subcutaneous) and rectal paracetamol (250 mg/kg; GlaxoSmithKline, Brentford, United Kingdom) were administered for overnight pain relief. Saline was administered intraperitoneally (2 × 1.5 mL) to replace fluid loses. Following surgery, animals were returned to their cages with free access to food and water. Cages were placed half over a heat mat to allow animals to thermoregulate during recovery.

### ICP Measurement

ICP (mmHg) was measured using a fibre-optic pressure sensor (Opsens Solutions, Quebec, Canada). Rats were fixed in a stereotaxic frame with ear bars in and a burr hole was made −1.8 mm lateral and −2 mm posterior to Bregma on the left parietal bone. A hollow polyether ether ketone (PEEK) screw (5 mm long, 1 mm internal diameter) was fixed in the burr hole. The screw was filled with saline and the pressure sensor wire was sealed 5 mm deep so that the sensor was sitting above the dura. Correct positioning was confirmed when a trace of pulse and respiratory oscillations were observed after the wire was sealed in place. Baseline ICP was recorded for 30 min prior to photothrombotic stroke, and average ICP was recorded from 17.5 to 18 h post-stroke in all animals. ICP was recorded up to 21 h in some animals but not all as experiment timing restricted our ability to correct displacements of the ICP probe that occurred after CSF tracer infusion.

### Photothrombotic Stroke

A light source (100,000 LUX, 5 mm diameter, Olympus Corporation LG-PS2, Tokyo, Japan) was placed over the right parietal bone 0.5 mm from the skull surface, 1.2 mm posterior to Bregma. While the light source was concentrated on the skull, Rose bengal in saline (0.01 g/kg; Sigma, St. Louis, MO, United States) was infused into the right femoral vein followed by 1 mL saline. The light source remained constant for 20 min after Rose bengal infusion. Sham animals received only saline + light exposure.

### Laminectomy

Laminectomy was carried out as previously described (Walker et al., 2015). Briefly, an incision was made from the back of the head caudally to the mid-thoracic region. The connective tissue was blunt dissected away and a cut was made through the midline of each muscle layer. The paraspinous muscles were retracted to expose the spinal column and connective tissue was blunt dissected. A dental drill (Saeshin Precision, Paho-dong, Korea) was used to thin the laminae of the C7 and T1 vertebrae. The laminae were then cut and removed at these points to expose the spinal cord.

### Evans Blue Dye Infusion

After stroke, two additional PEEK screws were fixed −1.8 mm lateral and −0.8 mm posterior to Bregma on each parietal bone to guide ventricle catheter penetration. The opening of the screw was sealed using caulking material (Silagum) prior to recovery of the animal after stroke and was only removed prior to lateral ventricle infusion 18 h post-stroke.

At 18 h post-stroke, a 27G neonatal lumbar needle (Becton, Dickinson and Company, NJ, United States) connected to PE-10 tubing (internal diameter 0.381 mm; SAI) and a syringe containing Evan’s blue dye (2% w/v in artificial CSF) was sealed 9 mm deep into each screw (5 mm screw length + 4 mm lateral ventricle depth) so that it would pierce the lateral ventricles. Evans blue dye was infused bilaterally into the lateral ventricles (20 μL total volume, 2 μL/min total with 1 μL/min in each ventricle). Infusion was controlled by a syringe driver (Harvard Apparatus, Holliston, MA, United States). Penetration of the ventricles was later confirmed by histology.

### Image Acquisition and Analysis of CSF Transit Speed

**FIGURE 1:**
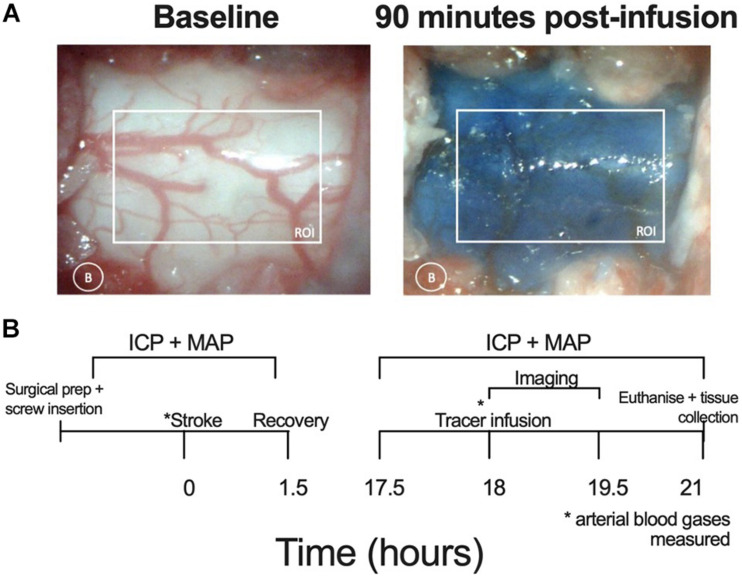
**(A)** Representative images showing the respective absence and presence of Evans blue dye at baseline and 90 min post-infusion at the C7-T1 region in a stroke animal without ICP rise. The white box represents the region of interest (ROI) for quantifying contrast and the white circle indicates lateral measurement of background (B) changes. **(B)** Experimental timeline. Lateral ventricle screw insertion and physiological variable recordings were carried out prior to stroke. Evans blue dye was infused 18 h post-stroke and imaging was carried out 0–90 min post-infusion. Post-stroke physiological variables were recorded from 17.5 to 21 h post-stroke. Deep cervical lymph nodes were extracted following euthanasia. ICP, intracranial pressure; MAP, mean arterial pressure, * time point at which arterial blood gases (ABG) measured.

White light images were acquired using a microscopic eyepiece (DEM130, CMOS chip, Scopium) connected to a stereo microscope (Olympus SZX7). The eyepiece was connected to a PC running pre-installed image acquisition software. An image was captured every minute for 90 min. These images were then loaded into ImageJ as a sequence. The images were converted to an 8-bit format with the colour channels split; only the red channel was used for analysis as this minimized contrast from blood vessels. Two regions of interest were selected: the spinal cord and a small region of tissue lateral to the spinal cord to correct for background noise. Difference between each image and baseline were calculated to show contrast development (Evans blue dye build up at the C7-T1 CSF space). Values for 100% maximum contrast were determined as the maximum contrast observed throughout the imaging period and this was used to determine 50% maximum contrast. Average area under the curve (AUC) of contrast development and time taken to reach 50% maximum contrast (T50%max) were used as an indicator of spinal CSF transit speed. Contrast development at the spinal CSF space is shown in Figure 1A.

### Quantification of Evans Blue Dye in Deep Cervical Lymph Nodes

The first bilateral deep cervical lymph nodes of each animal were removed following sacrifice and were frozen at −80°C until formamide extraction. Formamide extraction of Evans blue dye from the lymph node tissue was carried out as previously described but with some minor modifications (Krzyzanowska et al., 2010). Briefly, deep cervical lymph nodes were removed from the freezer and thawed. Once thawed, samples were dried in a 56°C oven for 1.5 h. Dried samples were added to an Eppendorf containing 50 μL of formamide, care was taken to ensure the entire tissue sample was submerged. The Eppendorfs containing both sample and formamide were placed back into the 56°C oven for 48 h to allow for Evans blue dye extraction into the solution.

Following extraction of Evans blue dye from the deep cervical lymph nodes into formamide, each sample was placed into a well of a 96-well plate, which contained three rows of standard curve solution (starting concentration 128 μg/mL formamide with a 1:2 dilution for each step, ending with 0.5 μg/mL formamide). Absorbance was determined at 620 nm in a spectrophotometer (SPECTROstar Nano plate reader, BMG Labtech) and the standard curve was used to determine concentration from absorbance within the program. Concentration of Evans blue dye in both the ipsilateral and contralateral deep cervical lymph nodes was assessed and these were averaged to obtain a single value for each animal.

### Histological Analysis

Animals were euthanised at 3 h post-infusion and transcardially perfused with 0.9% saline. The brains were then fixed in neutral-buffered formalin before being processed and paraffin embedded. Coronal sections of 5 and 10 μm were cut and stained with haematoxylin and eosin. Images were scanned using a digital slide scanner (Aperio Technologies, Vista, CA, United States) and image analysis was carried out within the Aperio program. Image analysis was confirmed by an independent investigator and any cases with >10% discrepancy were flagged for review. Infarct volume (corrected for oedema) was determined by subtracting the measured interhemispheric volume difference (oedema volume, ipsilateral–contralateral) from the measured infarct volume for each slice.

### Experimental Design

The experimental timeline is outlined in Figure 1B. Physiological variables were recorded at baseline, prior to photothrombotic stroke and at 18 h post stroke, prior to dye infusion. Screws were placed over the lateral ventricles and a dental cap was fitted after photothrombotic stroke. A C7-T1 laminectomy was performed at 17.5 h post-stroke to expose this area of the spinal cord. Bilateral infusion of 10 μL (1 μL per minute) Evans blue dye into both lateral ventricles (20 μL total) took place at 18 h post-stroke. Cisterna magna injection was not possible due to restricted access with the imaging set-up. White light images were acquired of the C7-T1 spinal cord every minute from 0 to 90 min post-infusion and images were analysed using ImageJ software (NIH). The animals were sacrificed at 3 h post-infusion and deep cervical lymph nodes were extracted and frozen at −80°C until formamide extraction and spectrophotometric analysis took place.

We previously reported a transient ICP rise that peaks 20–22 h post-stroke (Murtha et al., 2015; Beard et al., 2016). In this experiment, we chose 18 h post-stroke as our time point for this investigation. This is because we hypothesised that changes to CSF flow would be present at this earlier time point, which would contribute to the ICP rise we observe later. If we investigated CSF changes at 20–22 h, there is a high likelihood of missing these changes, as we would expect the system to begin to return to baseline levels at this point.

Our initial experiments demonstrating ICP rise utilised the MCAo model of stroke in rats (Murtha et al., 2014a, 2015). We and others have shown that MCAo can cause damage to the choroid plexus, which may influence CSF volume (Ennis and Keep, 2006; Beard et al., 2016). We aimed to pursue a line of investigation (contributions of CSF to ICP rise) using a rat model of stroke that does not cause choroid plexus damage. We have shown in two studies that ICP rise also occurs after photothrombotic stroke (Beard et al., 2016; Patabendige et al., 2019). Therefore, we chose to continue using this model for our study. Use of photothrombotic stroke also minimises surgical intervention, particularly around the cervical region, to prevent interference with lymphatic vessels.

### Statistical Analysis

Sample size calculations based on pilot data were conducted on G^∗^Power 3.1.9.2 and indicated that five stroke animals and five sham animals were required to detect a 30% change in CSF tracer transit between stroke and sham animals with 80% power and α error probability of 0.05 (*SD* = 6.2). The bimodal distribution of stroke animals with and without ICP rise prompted us to increase the number of animals so that we could compare changes to CSF flow in the presence and absence of ICP rise.

Statistical analyses were carried out using GraphPad Prism 7 (GraphPad Software, La Jolla, CA, United States). Data were tested for normal distribution using a D’Agostino and Pearson normality test. Comparisons between two groups were analysed by unpaired Student’s *t*-test for normally distributed data and by Mann-Whitney *U* test for non-normally distributed data. Area under the curve analysis was used to determine differences in contrast development over time. These values were presented as mean ± standard error of the mean (SEM) to allow for a comparison of average differences observed at each time point.

Additional statistical analyses were conducted *post hoc* to more accurately determine the association of spinal CSF flow and cervical lymphatic tracer transit with ICP elevation, since some stroke animals did not have a significant ICP rise. In these analyses, comparisons between three or more groups were carried out using one-way ANOVA for normally distributed data and using a non-parametric Kruskal-Wallis test for non-normally distributed data. A *post hoc* Bonferroni test was conducted after ANOVA and a Dunn’s test was conducted after Kruskal-Wallis test to identify differences between groups. All normally distributed data values are presented graphically as average ± standard deviation (SD) and non-normally distributed data as median and interquartile range (IQR), 25 and 75% percentile, unless otherwise stated. Relationships were determined by Pearson correlation for normally distributed data and Spearman correlation for non-normally distributed data.

## Results

### Physiological Parameters

Physiological parameters measured at baseline and 18 h post-stroke are presented for each group in Table 1.

**TABLE 1:** Comparison of physiological parameters between baseline and 18 h post-stroke within each group, paired *t*-test.

	Stroke with ICP rise		Stroke without ICP rise		Sham	
	Baseline	18 h	Baseline	18 h	Baseline	18 h
Respiratory rate (per minute)	62 ± 3	60 ± 3*^†^	68 ± 6	69 ± 6	64 ± 3	69 ± 7
Heart rate (BPM)	436 ± 20	421 ± 33	453 ± 10^†^	427 ± 34	428 ± 12^†^	414 ± 26
Mean arterial pressure (mmHg)	92.9 ± 4.8	90.4 ± 5.4	90 ± 9.2	98.5 ± 6.4^††^	87.4 ± 7	87.2 ± 6.4
SpO_2_ (%)	97.9 ± 1.0	98.0 ± 2.4	96.7 ± 2.9	97.8 ± 2.6	98.7 ± 1.1	98 ± 2.5
paO_2_ (mmHg)	185 ± 23 (*n* = 6)^†^	200 ± 25 (*n* = 6)	164 ± 28	193 ± 40	148 ± 23 (*n* = 9)	196 ± 22 (*n* = 9)***
paCO_2_ (mmHg)	61.2 ± 8.2 (*n* = 6)	62.2 ± 9.8 (*n* = 6)	52.3 ± 9.2^†^	60.4 ± 5.9	63.5 ± 5.5 (*n* = 9)	61.6 ± 7.4 (*n* = 9)**
pH	7.28 ± 0.03 (*n* = 6)	7.32 ± 0.03 (*n* = 6)	7.31 ± 0.03^†††^	7.32 ± 0.02	7.25 ± 0.01 (*n* = 9)	7.3 ± 0.04 (*n* = 9)*

Comparison between stroke with ICP rise (n = 7), stroke without ICP rise (n = 5), and sham (n = 10) at baseline and 18 h post-stroke, one-way ANOVA with post hoc Bonferroni test (stroke groups vs. sham). *p ≤ 0.05. **p ≤ 0.01. ***p ≤ 0.001, paired t-test of baseline vs. 18 h post-stroke. ^†^p ≤ 0.05. ^††^p ≤ 0.01. ^†††^p ≤ 0.001, one way ANOVA with post hoc Bonferroni test of stroke with/without ICP rise vs. sham. BPM, beats per minute.

### Exclusions

All excluded animals were from the sham group.

Three animals were excluded from experimentation as they presented with congenital deformities to the spinal column that prevented laminectomy. Four animals were excluded due to errors in surgical placement of screws or problems with infusion, and one animal died before imaging was complete. One sham animal received Evans blue dye infusion, but it was later found that ventricular penetration did not take place due to misalignment of screws, this animal was excluded from the analysis. All stroke animals demonstrated tissue infarction; therefore, none were excluded.

### There Was a Bimodal Distribution of Stroke Animals With and Without ICP Rise

**FIGURE 2:**
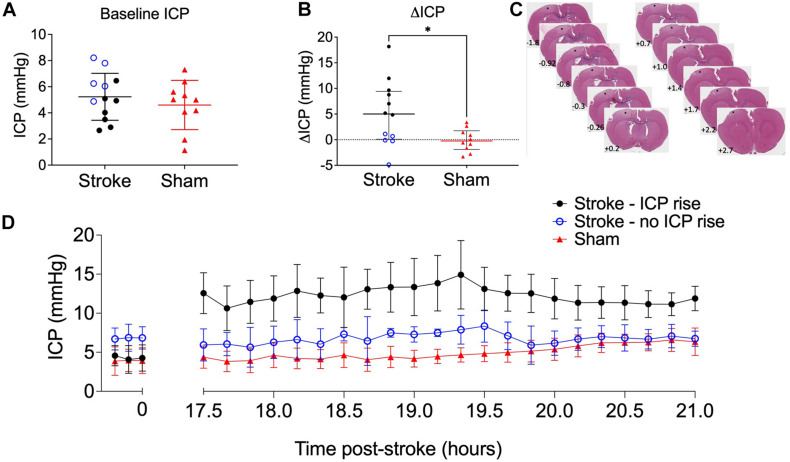
**(A)** Baseline intracranial pressure (ICP; mmHg) in stroke (*n* = 12; black closed circles and blue open circles) and sham (*n* = 10; red triangles) rats, Student’s *t*-test. **(B)** Change in ICP from baseline to 18 h post-stroke in stroke rats and in sham rats. ^∗^*p* ≤ 0.05, Mann-Whitney *U* test presented as median and interquartile range. Blue open circles indicate animals that did not have an ICP rise above +4.4 mmHg. All animals were included in statistical analysis. **(C)** Representative image of cortical ischaemia after H&E staining, Bregma +2.7 to −1.8. ^∗^ indicates infarct. **(D)** Temporal profile of mean ICP at baseline and between 17.5 and 21 h post-stroke. Nb. For all graphs stroke rats with ICP rise greater than +4.4 mmHg (*n* = 5; black circles), stroke rats without ICP rise (*n* = 5; blue open circles), and sham rats (*n* = 7; red triangles).

Baseline ICP was not significantly different between stroke (5.23 ± 1.8 mmHg, *n* = 12) and sham (4.6 ± 1.88 mmHg, *n* = 8) groups, *t*(20) = 0.79, *p* = 0.44 (Figure 2A). We used + 4.4 mmHg as the determinant of ICP rise (baseline to 18 h) as it represents twice the standard deviation of ΔICP of sham animals. ICP rose significantly in stroke animals (5.0 mmHg, IQR = 0.1–9.4, *n* = 12) but not in sham animals (−0.3 mmHg, IQR = −1.9 to 1.7, *n* = 10; *U* = 27, *p* = 0.03; Figure 2B) from baseline to 18 h post-stroke. There was a bimodal distribution of stroke animals with and without ICP rise with 7/12 stroke animals showing ICP rise greater than +4.4 mmHg between baseline (pre-photothrombotic stroke) and 18 h post-stroke. For subsequent analyses to determine whether there was an association between ICP rise and CSF tracer outflow, we separated stroke animals into two groups depending on whether they had an ICP rise or not. Average infarct volume was 8.67 ± 4.8 mm^3^. Tissue infarct is shown in Figure 2C. ICP change between baseline and 17.5–21 h post-stroke is shown for stroke rats with ICP rise (*n* = 5), stroke rats without ICP rise (*n* = 5), and sham rats (*n* = 7) in Figure 2D.

### Oedema Is Not the Cause of ICP Rise 18 h After Photothrombotic Stroke

**FIGURE 3:**
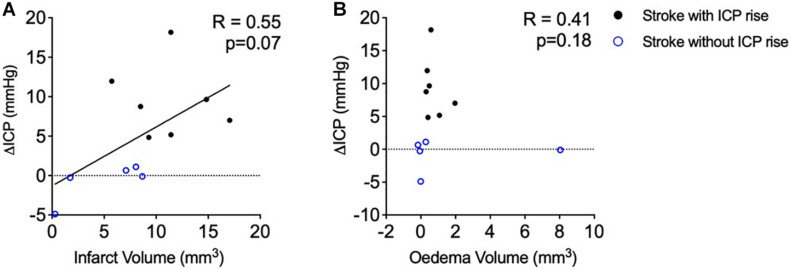
**(A)** Relationship between infarct volume (mm^3^) and intracranial pressure rise (ΔICP; mmHg). Black closed circles represent stroke animals with an ICP rise greater than +4.4 mmHg (*n* = 7) and blue open circles represent those without ICP rise (*n* = 5). **(B)** Relationship between oedema volume (mm^3^) and ΔICP. A Spearman correlation was used for analyses. Black closed circles represent stroke animals with an ICP rise greater than +4.4 mmHg (*n* = 7) and blue open circles represent those without ICP rise (*n* = 5).

There was no significant correlation between infarct volume and ΔICP from baseline to 18 h post-stroke (Figure 3A), *R* = 0.55, *p* = 0.07, Spearman’s correlation.

Oedema was not correlated with ΔICP between baseline and 18 h post-stroke (Figure 3B), *R* = 0.41, *p* = 0.18, Spearman’s correlation. Average oedema volume was 1.12 ± 2.26 mm^3^.

### Cranial Clearance of Evans Blue Dye to the Deep Cervical Lymph Nodes Was Reduced in Stroke Animals but Was Not Correlated With ΔICP

**FIGURE 4:**
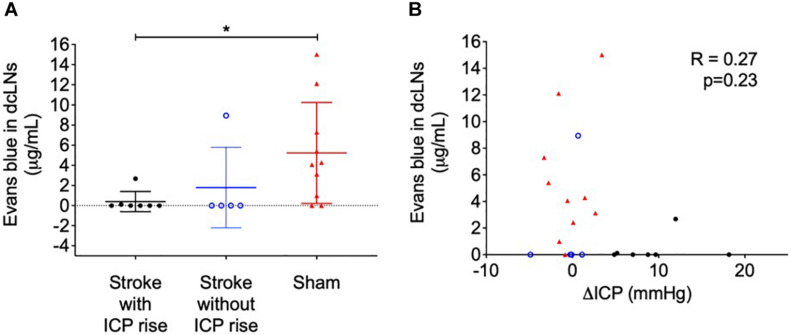
**(A)** Concentration of Evans blue dye extracted into formamide (μg/mL) from deep cervical lymph nodes (dcLNs) of stroke animals with intracranial pressure (ICP) rise greater than +4.4 mmHg (black closed circles), stroke without ICP rise (blue open circles), and sham (red triangles) animals. *p ≤ 0.05, Kruskal-Wallis test with *post hoc* Dunn’s test between groups. **(B)** Relationship between ΔICP (mmHg) and the concentration of Evans blue dye in dcLNs. A Spearman correlation was used for analyses.

Evans blue dye concentration was not significantly different between ipsilateral and contralateral lymph nodes for all groups (2.74 ± 4.76 μg/mL formamide vs. 3.08 ± 4.53 μg/mL formamide), *t*(42) = 0.24, *p* = 0.81. Therefore, we averaged ipsilateral and contralateral values to obtain one value per animal to compare between groups. The concentration of Evans blue dye extracted from the deep cervical lymph nodes was significantly different between groups, *p* = 0.02 (Figure 4A). Stroke animals with ICP rise (0 μg/mL formamide, IQR = 0–0.11) were similar to stroke animals without ICP rise (0 μg/mL formamide, IQR = 0–4.47), and both had a lower concentration when compared to sham animals (4.17 μg/mL formamide, IQR = 0.74–8.51). A *post hoc* Dunn’s test found a significant difference between sham animals and stroke animals with ICP rise, *p* = 0.03, but no significant difference between sham animals and stroke animals without ICP rise, *p* = 0.1.

Cervical lymph node concentration of Evans blue dye was not correlated with ΔICP, *R* = 0.27, *p* = 0.23 (Figure 4B), infarct volume, *R* = −0.19, *p* = 0.55, or oedema volume, *R* = −0.22, *p* = 0.5.

### Spinal CSF Transit Speed Was Correlated With ICP Rise

**FIGURE 5:**
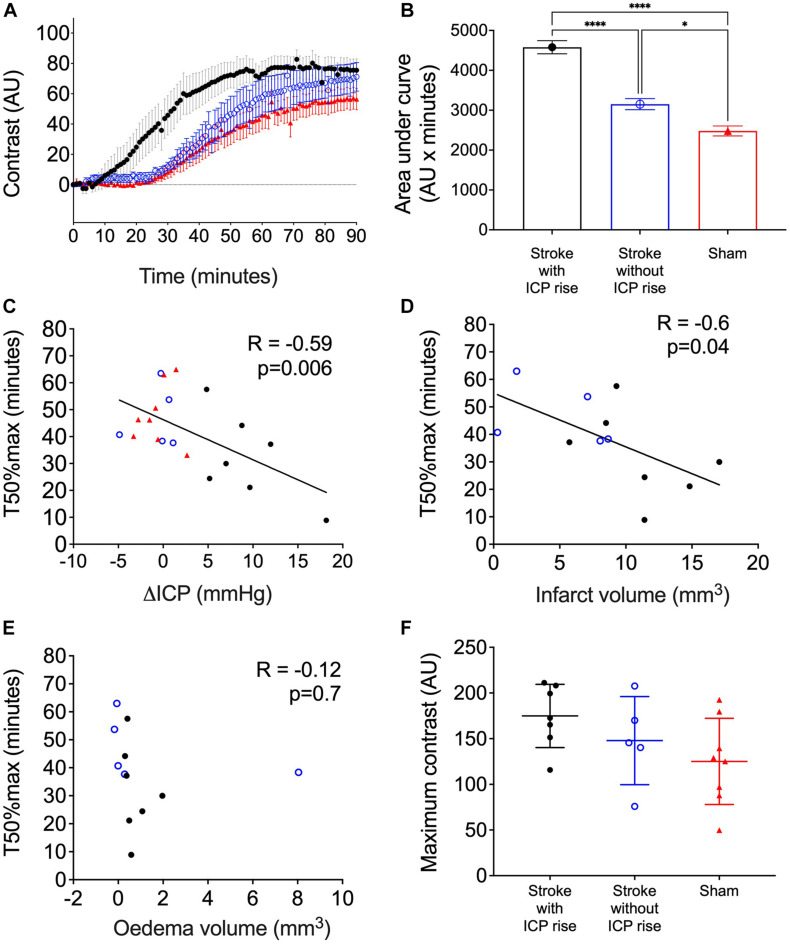
**(A)** Average contrast development (arbitary units; AU) over time (minutes) for stroke rats with intracranial pressure (ICP) rise greater than +4.4 mmHg (black closed circles), stroke rats with no ICP rise (blue open circles), and sham rats (red triangles). Presented as mean ± standard error of the mean (SEM). **(B)** Area under the curve (AU × minutes) presented for each group as mean ± SEM, ANOVA with *post hoc* Bonferroni test. **(C)** Relationship between ΔICP (mmHg) and time to reach 50% maximum contrast (T50%max; minutes). A Spearman correlation was used to determine the relationship. **(D)** Relationship between infarct volume (mm^3^) and T50%max, Pearson correlation. **(E)** Relationship between oedema volume (mm^3^) and T50%max, Pearson correlation. **(F)** Maximum contrast (AU) observed in each group, mean ± standard deviation.

Average contrast development (Arbitrary Units; AU) ± SEM over time (minutes) for each group is shown in Figure 5A. AUC of contrast development was greater in stroke animals with ICP rise (mean = 4,579, SEM = 164 AU × minutes, *n* = 7), compared with stroke animals without ICP rise (mean = 3,152, SEM = 138.4 AU × minutes, *n* = 5), and sham animals (mean = 2,481, SEM = 124.9 AU × minutes, *n* = 8), *F*(2, 17) = 59.8, *p* < 0.0001 (Figure 5B). A *post hoc* Bonferroni test confirmed a significant difference between stroke animals with ICP rise and sham animals [*p* < 0.0001, *t*(17) = 10.82], and stroke animals without ICP rise and sham animals [*p* = 0.02, *t*(17) = 3.14].

There was a significant inverse correlation between ΔICP and T50%max. Animals with a higher ΔICP reached 50% maximum contrast faster than those with a lower ΔICP, *R* = −0.59, *p* = 0.006, Spearman’s correlation (Figure 5C).

### Infarct Volume but Not Oedema Correlates With Faster Spinal CSF Transit

There was a significant inverse correlation between infarct volume and T50%max, which demonstrates faster transit of Evans blue dye to the C7-T1 spinal subarachnoid space in animals with larger infarct volumes (Figure 5D), *R* = −0.6, *p* = 0.04.

Oedema volume was not correlated with T50%max (Figure 5E), *R* = −0.12, *p* = 0.7.

### Maximum Change in Contrast Was Not Different Between Groups

The maximum change in contrast from 0 to 90 min post-infusion did not significantly differ between stroke animals with ICP rise (174.9. ± 34.6 AU, *n* = 7), stroke animals without ICP rise (147.9 ± 48.2 AU, *n* = 5), and sham animals (125.1 ± 47.15 AU, *n* = 8), *F*(2, 17) = 2.45, *p* = 0.11 (Figure 5F).

### A Correlation Between Spinal CSF Transit Speed and Clearance to the Deep Cervical Lymphatics Could Not Be Determined

**FIGURE 6:**
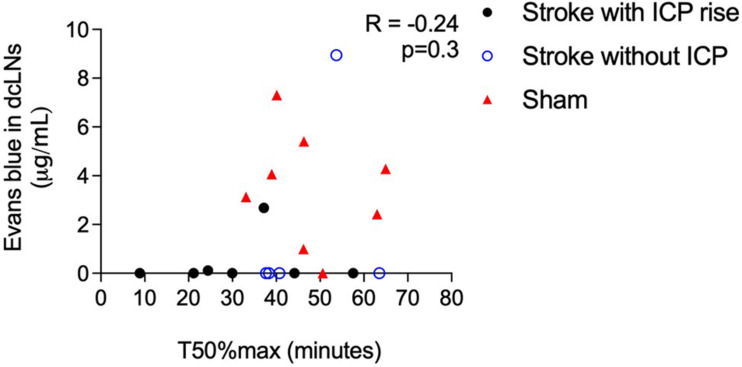
Correlation between speed of CSF transit to the spinal subarachnoid space (T50%max) and concentration of Evans blue dye in deep cervical lymph nodes (dcLNs; μg/ml) in stroke animals with intracranial pressure (ICP) rise greater than +4.4 mmHg (black closed circles), without ICP rise (blue open circles), and sham animals (red triangles). A Spearman correlation was used for analyses.

Cranial CSF clearance to the deep cervical lymphatics was reduced in stroke animals with and without ICP rise and spinal CSF transit speed was faster in animals with ICP rise (Figure 6). There was no correlation between these variables, *R* = −0.24, *p* = 0.3.

## Discussion

We previously found an oedema independent ICP rise that peaks 20–22 h after experimental ischaemia. Here, we report that it can be detected as early as 18 h. We showed that clearance of cranial CSF tracer into the deep cervical lymphatics is reduced, and there is faster movement of tracer into the spinal subarachnoid space 18 h after photothrombotic stroke in rats. Prior work has shown resistance to CSF outflow is increased after experimental ischaemia (Patabendige et al., 2019; Alshuhri et al., 2020). This work suggests that cranial clearance of CSF is reduced, which may explain increased outflow resistance. Further, we found impaired cranial clearance in animals with and without ICP rise. Animals with increased ICP had increased spinal CSF transit speed, suggesting this pathway is utilised as a compensatory mechanism when both cranial clearance is reduced and ICP is elevated.

All but two stroke animals had no Evans blue dye in their deep cervical lymph nodes after intraventricular infusion and 3-hour circulation. This indicates a significant disruption of CSF clearance 18 h after stroke. Resistance to CSF outflow in the spine is greater than resistance to CSF outflow in the cranium (Bozanovic-Sosic et al., 2001). Therefore, when cranial clearance pathways are impaired, we would expect an overall increase in resistance to CSF outflow across the whole cranio-spinal compartment. We previously demonstrated this increased resistance to CSF outflow 18 h after photothrombotic stroke in rats (Patabendige et al., 2019) and another group reported similar findings 24 h after MCAo (Alshuhri et al., 2020). The current results indicate that reduction in cranial CSF clearance may contribute to increased resistance.

**FIGURE 7:**
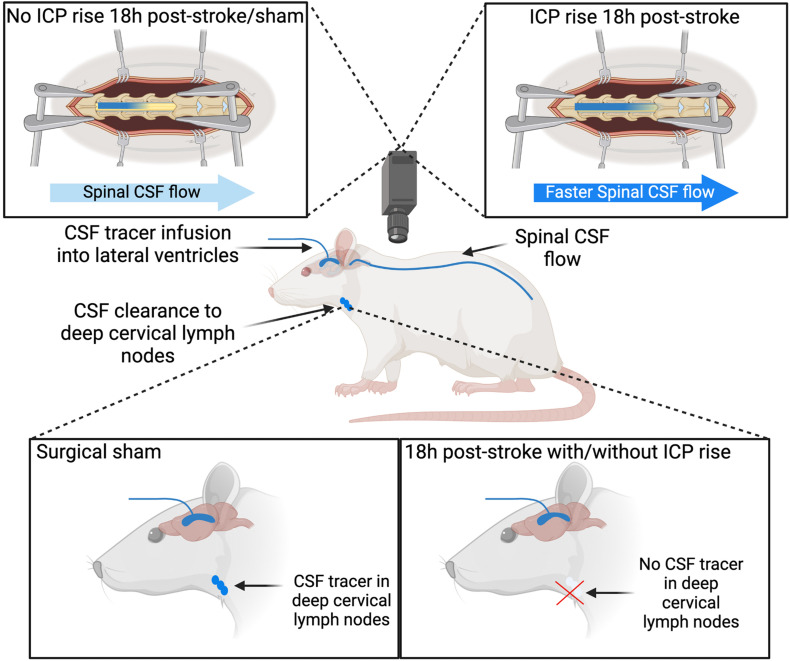
Cerebrospinal fluid (CSF) tracer movement into the deep cervical lymph nodes and spinal CSF space. Stroke animals have impaired CSF clearance to deep cervical lymph nodes 18 h post-stroke with and without intracranial pressure (ICP) rise. Increased ICP 18 h post-stroke is associated with faster CSF tracer movement into the spinal CSF space, compared to stroke animals without ICP rise and sham animals.

Impaired CSF clearance to the deep cervical lymphatics was previously shown in response to neurological injury (Bolte et al., 2020). Therefore, we hypothesised that this pathway would provide insight into the underlying mechanisms of ICP rise 18 h post-stroke. The almost complete reduction of CSF clearance to deep cervical lymph nodes in stroke animals likely impeded our ability to detect a correlation with ICP rise, since that distribution was non-linear (Figure 4B). Animals without ICP rise still had impaired cranial CSF clearance. These observations may be explained by the time point of our experiments. Here, we aimed to capture changes to CSF dynamics and ICP at 18 h post-stroke but we previously reported peak ICP rise between 20 and 22 h (Beard et al., 2016). ICP values in this current study were smaller than those previously reported, due to the earlier time point of our experiments (Murtha et al., 2015; Beard et al., 2016). We expect that ICP would have continued to rise and peak at 20–22 h post-stroke. We speculate that disruptions to CSF flow may pre-empt ICP rise, which means it may be possible that the disruption to CSF flow we observed in the absence of ICP rise could be indicative of a delayed ICP rise that we did not capture due to our experimental timing. Despite these observations, we found that increased ICP was associated with increased spinal CSF transit. This suggests that movement of cranial CSF along this pathway is utilised as a compensatory mechanism in response to elevated ICP when direct cranial clearance pathways are absent (Figure 7). Similar observations of increased ICP, reduced cranial clearance, and increased spinal clearance of CSF were previously reported after subarachnoid haemorrhage related hydrocephalus in rabbits (Griebel et al., 1989). Further, previous work found that sealing of the cribriform plate in dogs prevented CSF tracer flow into the deep cervical lymphatics and instead promoted tracer accumulation in lymphatic vessels associated with spinal CSF drainage (Galkin, 1930). Recently, Proulx’s group demonstrated a predominant role for lymphatics at the sacral spine in spinal CSF clearance, although the proportion of CSF clearance at this site was minimal relative to that at the cervical lymphatics (Ma et al., 2019). We did not examine the sacral lymphatics in this study, but our current findings suggest that future investigations of CSF clearance post-stroke should include examination of spinal lymphatics to understand its contribution to overall clearance of CSF from the cranio-spinal compartment.

Our findings that oedema volume did not correlate with either ΔICP or T50%max are consistent with our previous report that oedema does not correlate with ICP rise 24 h after transient MCAo, and with work showing similar observations after permanent MCAo (Murtha et al., 2015; Alshuhri et al., 2020). Oedema volumes were assessed early and would likely be greater at later time points. However, we still observe ICP rise from 18 to 21 h, which suggests that mechanisms other than oedema contribute to ICP rise after photothrombotic stroke. We also found that ICP rise did not significantly correlate with infarct size, which is in line with our previous observations that ICP rise at 24 h is not an indicator of large infarct volume (Murtha et al., 2015; Beard et al., 2016). This is in contrast to ICP rise observed after large infarct 3–5 days post-stroke in animal models (Kotwica et al., 1991; Silasi et al., 2009) and in humans (Schwab et al., 1996).

In this study, we infused the CSF tracer, Evans Blue dye, directly into the lateral ventricles of rats (Boulton et al., 1999; Maloveska et al., 2018; Norwood et al., 2019). This type of intervention has the potential to disturb normal CSF dynamics; however, this technique is widely used and we took care to limit the infusion rate to lower than that of CSF production (normally 2.66–2.84 μL/min in rats) to maintain the integrity of the system as much as possible (Chiu et al., 2012). We also monitored ICP during infusion to ensure there were no significant changes. We used the low molecular weight, lipophobic tracer, Evans blue dye, for our studies to investigate cranial clearance of CSF since tracer movement differs depending on molecular size and weight (Yang et al., 2013), and we wished to approximate movement of fluid, rather than larger molecular weight, and potentially immunogenic, proteins within the fluid. No Evans blue dye was present (i.e., blue staining) around the infarct or surrounding brain tissue, which indicates that extravasation of CSF tracer to blood *via* the area of stroke due to blood-brain barrier breakdown is unlikely. Our choice of anaesthetic was advantageous to our study of CSF clearance. Anaesthetic-related hypercapnia was previously shown to impair glymphatic flow after intraparenchymal injection of tracer, while lymphatic clearance remained unaltered after direct infusion into CSF (Goodman and Iliff, 2020). This means that there was likely reduced parenchymal uptake of tracer in our study, allowing our findings to be largely representative of bulk CSF clearance pathways. Reduced paCO_2_ can reduce ICP by inducing vasoconstriction of the cerebral resistance arterioles, which reduces cerebral blood volume (Godoy et al., 2017). Stroke rats without ICP rise had a significantly lower baseline paCO_2_, and consequently reduced pH, when compared to sham animals in this study, although animals did not reach levels of hypocapnia (<24.75 mmHg) (Young et al., 1991). This is unlikely to explain the absence of ICP rise in this group as paCO_2_ was similar between all groups 18 h post-stroke. Further, if our observations of ICP were explained solely by CO_2_ then we would expect a greater ICP rise in this group as animals had a larger increase in paCO_2_ from baseline compared to other groups. We utilised photothrombotic stroke as the preferred model for this study to maintain choroid plexus integrity throughout our experiments. MCAo reduces blood flow to the choroid plexus by around 62% and causes choroidal oedema leading to reduced blood-CSF barrier integrity and increased CSF secretion (Ennis and Keep, 2006). Histology confirmed that ischaemia was restricted to the cortex in this current study.

It is important to consider the differences in CSF clearance between humans and animal models. Animal studies demonstrating CSF clearance pathways are relatively abundant when compared to human studies, largely due to their invasive nature. However, evidence of lymphatic drainage in humans was previously found in cadavers and imaging studies have shown CSF tracer movement into the nasal mucosa and deep cervical lymphatics of humans (de Leon et al., 2017; Eide et al., 2018; Proulx, 2021). It is important to remember that we have not yet arrived at a consensus regarding the relative proportion of CSF clearance attributed to lymphatic outflow pathways in humans. Nevertheless, the consequences of impaired CSF clearance are multi-faceted, potentially contributing to increased ICP, reduced cerebral blood flow, protein accumulation and aggregation, and cognitive decline (Griebel et al., 1989; Bolte et al., 2020).

## Conclusion

In this study we have shown that CSF tracer clearance into the cervical lymphatics is impaired 18 h post-stroke in a rat photothrombotic stroke model, and there is a compensatory increase in CSF transit to the spinal subarachnoid space. Taken with previous observations of increased resistance to CSF outflow after experimental ischaemia, we believe changes to CSF clearance post-stroke may contribute to changes in the regulation of ICP.

## Data Availability Statement

The raw data supporting the conclusions of this article will be made available by the authors, without undue reservation.

## Ethics Statement

The animal study was reviewed and approved by the University of Newcastle Animal Care and Ethics Committee.

## Author Contributions

SB carried out the surgical components of the study, analysed and interpreted the data, performed statistical analysis, and drafted the manuscript. DO and RH helped with interpretation of the data and manuscript drafting. D-GP and DO helped with the histology and image analysis. SA helped with the deep cervical lymph node extraction and subsequent quantification of Evans blue dye. NS and AP participated in the concept and design of the study, helped with interpretation of the data, and drafting the manuscript. All authors read and approved the final manuscript.

## Conflict of Interest

The authors declare that the research was conducted in the absence of any commercial or financial relationships that could be construed as a potential conflict of interest.

## Publisher’s Note

All claims expressed in this article are solely those of the authors and do not necessarily represent those of their affiliated organizations, or those of the publisher, the editors and the reviewers. Any product that may be evaluated in this article, or claim that may be made by its manufacturer, is not guaranteed or endorsed by the publisher.

## Abbreviations

ICP: Intracranial pressure
CSF: Cerebrospinal fluid
IQR: Interquartile range
MCAo: Middle cerebral artery occlusion
SD: Standard deviation
SEM: Standard error of the mean
dcLN: Deep cervical lymph nodes.
